# Intermediate care in nursing home after hospital admission: a randomized controlled trial with one year follow-up

**DOI:** 10.1186/1756-0500-7-889

**Published:** 2014-12-09

**Authors:** Jo Kåre Herfjord, Torhild Heggestad, Håkon Ersland, Anette Hylen Ranhoff

**Affiliations:** County Governor of Hordaland, Postboks 73105020, Bergen, Norway; Haukeland University Hospital, Jonas Lies vei 63, 5021 Bergen, Norway; Department of Clinical Science, University of Bergen and Kavli Research Centre for Geriatrics and Dementia, Haraldsplass Hospital, Ulriksdal 8, 5009 Bergen, Norway

**Keywords:** Acute care, Community services, Intermediate care, Elderly, Older people, Randomized controlled trial

## Abstract

**Background:**

Intermediate care is intended to reduce hospital admissions and facilitate early discharge. In Norway, a model was developed with transfer to intermediate care shortly after hospital admission. Efficacy and safety of this model have not been studied previously.

In a parallel-group randomized controlled trial, patients over 70 years living at home before admission were eligible if clinically stable, without need for surgical treatment and deemed suited for intermediate care by attending physician. Intervention group patients were transferred to a nursing home unit with increased staff and multidisciplinary assessment, for a maximum stay of three weeks. Patients in the control group received usual care in hospital. Blinding to group assignment was not possible.

The primary outcome was number of days living at home in a follow-up period of 365 days. Secondary outcomes were mortality, hospital admissions, need for residential care and home care services. Data were obtained from patient records and registers.

**Results:**

376 patients were included, 74 % female and mean age 84 years. There was no significant differences between intervention (n = 190) and control group (n = 186) for number of days living at home (253.7 vs 256.5, p = 0.80) or days in hospital (10.4 vs 10.5, p = 0.748). Intervention group patients spent less time in nursing home (40.6 days vs. 55.0, p = 0.046), and more patients lived independently without home health care services (31.6 % vs 19.9 %, p = 0.007).

For orthopaedic patients (n = 128), mortality was higher in the intervention group; 15 intervention patients and 7 controls died (25.1 % vs 10.3 %, p = 0.049). There was no significant difference in one-year mortality for medical patients (n = 150) or the total study population.

**Conclusions:**

This model of rapid transfer to intermediate care did not significantly influence number of days living at home during one year follow-up, but reduced demand for nursing home care and need for home health care services. In post-hoc analysis mortality was increased for orthopedic patients.

**Trial registration:**

The trial was registered 26. July 2013 at Current Controlled Trials and assigned with registration number ISRCTN21608185.

## Background

Intermediate care is a broad term describing health care services designed to provide adequate care closer to home, while preventing hospital admissions, facilitating early discharge and supporting patients with long-term conditions. Examples are ‘Hospital at Home’, step-up and step-down care home beds, early supported discharge and residential/day rehabilitation [1–3]. Intermediate services are supposed to bridge a gap between primary care and hospital services, providing adequate care closer to home at a lower cost while saving hospital beds and transportation costs.

Because services often are intended for patients with chronic diseases or have defined age limits, a great part of users will be older people [1, 4]. As an alternative to hospital admission there are potential benefits for patients, but also potential risks. Level of staffing and equipment will be lower in an intermediate care setting compared to a hospital, while target patient populations will tend to have a higher risk for complications and adverse events because of old age, co-morbidity and frailty.

Intermediate care services are implemented in several countries, but there are few studies and limited data on efficacy and safety for different patient groups. Young and coworkers found that the introduction of a city-wide intermediate care service did not reduce long-term care or hospital use [5]. However, in a randomized controlled trial of post-acute care for older people in community hospitals, patients had greater independence after 6 months [6, 7].

Several review articles and meta-analyses evaluating intermediate care are inconclusive. A Cochrane review found that trials of early discharge hospital at home showed diverging results for readmission rates, need for residential care and cost savings. Evidence of economic benefit or improved health outcomes was insufficient [8].

A Cochrane review of intermediate care in nursing-led in-patient units found some evidence that fewer patients were discharged to institutional care, but a possibility of increased early mortality could not be discounted [9]. In an article evaluating different models of intermediate care for older people in the United Kingdom, Woodford and George found that outcomes typically were similar to traditional hospital care. Intermediate care services tended to be met with high patient satisfaction, yet there was no evidence that they reduced acute hospital use or that they were cost efficient [10]. Young concluded in a similar review that intermediate care in England has not yet fulfilled its expectations. With the exception of early supported discharge and hospital at home services for stroke and acute exacerbation of COPD, there appeared to be no net reduction in health service costs [11].

Health authorities in Norway have recently launched a new health care policy, ‘The Coordination Reform’ [12]. One of the key elements is a strong initiative for community-based intermediate care services. The rationale for this is partly based on studies from Trondheim, where Garåsen and coworkers found increased survival and less need for community-based health care services for patients treated in an intermediate care unit as compared to usual care in hospital [13, 14].

A 19-bed intermediate care unit was established at Storetveit nursing home in 2005 with the aim to provide post-acute treatment for elderly people within few days after acute admission to hospital. The project was a collaboration between the municipality if Bergen and the two hospitals serving the town (Haraldsplass Deaconness Hospital and Haukeland University Hospital).

Soon after the unit’s inauguration, a consulting firm (Agenda, in collaboration with COWI) was commissioned to organize and carry out an evaluation with regard to costs and benefits. They designed a parallel-group randomized study, aiming to assess costs and patients’ functional outcome and quality of life 3 months after treatment in the intermediate care unit. However, questionnaire response rates were low and information gathered from other sources was indeterminate. The investigators were on the whole unable to draw any decisive conclusions. A report from this first phase of the study is available online, albeit in Norwegian language only [15].

### Objective

We found the unresolved questions about this new model of intermediate care unsatisfactory, and that the rapid assessment and transfer of frail patients was not sufficiently studied. With approval from the regional ethics committee, we decided to continue the study with a second phase, involving further gathering of data from the same population and measuring a new set of endpoints. The objective was to evaluate efficacy and safety of this model of intermediate care with early transfer, compared to usual hospital treatment. In this article, we report the findings from the investigations in the second phase of the study.

## Methods

### Trial design

The trial design was a parallel-group study with balanced randomization 1:1.

### Eligibility criteria

Patients admitted acutely from home to medical or orthopaedic departments were eligible for participation if they were a resident of Bergen municipality, aged 70 years or older, respiratory and circulatory stable and deemed able to return home within three weeks. Exclusion criteria were severe dementia, delirium, any need for surgery or intensive care treatment. Patients in need of surgery or intensive care treatment could not be included in the study as these services were not provided at the intermediate care facility.

When inclusion started, doctors and nurses in involved hospital departments were informed about the study and criteria for selecting patients. Printed instructions were made available, but they did not specify how to identify or assess delirium or dementia. If in doubt, the attending physician could discuss patients with the geriatrician at the intermediate care unit. Patients considered to have mild or moderate dementia were eligible. Suitable patients were invited to participate in the trial if attending physician considered intermediate care an appropriate treatment option, and if randomization could take place within the first 72 hours after admission.

### Settings and locations

The setting of the study and data collection took place at Storetveit Nursing Home, Haraldsplass Deaconess Hospital and Haukeland University Hospital, all located in Bergen, Norway. These two hospitals handle all acute admissions from the city’s population of 260,000.

### Interventions

The description is structured according to the principles of the TIDieR checklist [16]. The essence of the intervention was a rapid transfer to intermediate care unit in a nursing home.

The underlying rationale is that studies have shown that some elderly patients can be successfully treated in a “step-down” facility at the end of a hospital stay. In previous studies, patients have typically been selected after several days in hospital. If transfer to intermediate care would be safe and feasible earlier in the course, the service could be extended to a larger group of patients and have a greater impact in saving health care costs.

The intervention did not incorporate physical or information materials, apart from the information sheet explaining the study given to eligible patients prior to consent. There were however several procedures specific for the intermediate care unit. A significant difference from previous models was the short time-frame before transfer. In the intervention group patients were examined and evaluated after a model of comprehensive geriatric assessment [17], and given a Barthel Index score. When appropriate, screening tools and scales including MMSE (Mini-mental state examination), Geriatric Depression Scale and Cornell Scale for Depression were applied. Diagnoses and treatment regimens were evaluated and adjusted. The list of prescribed medication was critically evaluated and checked for harmful interactions, using the Norwegian internet database http://www.interaksjoner.no/. The facility did not have access to radiologic examinations, but some blood tests could be analyzed on the spot (hemoglobin, C-reactive protein, glucose, prothrombin time/INR). Other blood samples were sent to the hospital lab for analysis within a few hours.

Patients were mobilized out of bed and out of the room as soon as possible, and were encouraged to practice and maintain daily self-care activities and to exercise individually indoors and outdoors when possible. They were offered individual physiotherapy and group-based exercise, and were equipped with necessary mobility aids like crutches, walkers or wheelchairs. The overall aim was maximum independence, enabling patients to resume living at home.

Nutrition status was evaluated, with special awareness for patients with risk of undernutrition or malnutrition. There was a general emphasis on positive atmosphere at meal times, inviting surroundings and appetizing presentation. Individual adjustments were made when appropriate regarding composition of nutrients, dietary supplements, meal times or number of meals. Information about patients’ home situation and caregivers were obtained. If necessary, the staff assisted patients to apply for further home health care services or residential care, and could also refer patients to occupational therapist or speech therapist.

The services were provided by a multi-disciplinary team of physician, nurse, physiotherapist and health care worker. The residing physician would either be a specialist in geriatric medicine and internal medicine (consultant), or a junior doctor (trainee) supervised by the geriatrician. Skilled nurses and health care workers were on duty at all times. Physician and physiotherapist were present every workday.

The intervention was delivered in the setting of an intermediate care unit located in a nursing home, separate from the hospitals. The unit consisted of a single ward with 15 beds. Prior to the study, the ward was refurbished and modified to accommodate higher level of staffing, drug storage and medical and laboratory equipment. Number of full-time nurse positions in particular was increased, from 3 to 12.7. The unit was supplied with equipment for intravenous treatment, nebulizer for inhalation, ECG, bladder scan, pulse oximetry and oxygen supply.

Observation, mobilization, nutrition and practicing self-care were addressed on a daily basis by nurses and health care workers. Physician and physiotherapist examined each patient within the first working day after admission. The doctor made a ward round at least twice a week for each patient and other team members participated in the pre-ward round briefing. After initial assessment, the physiotherapist gave individual treatment to selected patients and organized group exercise for all patients three times a week. The multi-disciplinary team met twice weekly, discussed patients systematically and decided further plans for treatment. This included decisions regarding time of discharge within the 3-week maximum and making arrangements for further treatment and care after discharge.

A few measures were to some extent tailored to patients’ individual needs. This would apply to quantity of individual physiotherapy, use of dietary supplements and length of stay. The intervention was not modified during the course of the study.

### Control group

Patients in the control group stayed in hospital and received usual care according to their condition. The exact contents of “usual care” could in principle vary between the two hospitals and between different departments in the same hospital, as there were no special requirements given regarding the details of hospital treatment. Some major differences between the intermediate care unit and the hospitals would be presence of physician at weekends, availability of diagnostic tests, especially radiologic examinations, and monitoring equipment like telemetry. In hospitals, multi-disciplinary assessment was not applied systematically and patients were not likely to meet a geriatrician.

### Outcome measures and assessment

The study has two distinct phases. Originally, the chosen primary outcome measures in the initial phase of the study were functional outcome and quality of life. At the same time, investigators attempted to evaluate costs for the two alternative treatment options. Over a three-month period patients were invited to answer several questionnaires, including self-report of health status, EQ-5D (EuroQoL) and visual analogue scale. Unfortunately, questionnaire response rates were low and supplementary information gathered from other sources was insufficient or difficult to interpret. The investigators were on the whole unable to draw any decisive conclusions. A report from this first phase of the study is available online, albeit in Norwegian language only [15].

In this article we report the second phase of the study. We decided to collect more data from the same patient group, and obtained approval by The Regional Committee for Medical Research Ethics for a prolonged observation period and new outcome measures. Our new pre-specified primary outcome was the number of days patients would be alive and living at home throughout the first year after randomization. Secondary outcome measures were mortality, days spent in hospital, days in nursing homes and use of home health care services.

In this study we define use of home health care services as any supportive care provided in the home by the publicly funded health care system. In a Norwegian setting virtually no providers of these services operate without public funding. By supportive care we mean help provided by licensed healthcare professionals, non-medical caregivers or care assistants for medical needs, help in activities of daily living and help for practical needs like cleaning the home and preparing meals.

Data were obtained from electronic patient registers and digital health records at Haukeland and Haraldsplass hospitals and community health care services in Bergen municipality. The two hospitals’ information and communication departments provided necessary printouts from registers, and one of the authors (JKH) assessed medical records and community registers for all patients. The acquired data included dates of admission and discharge from hospitals and nursing homes, diagnoses from hospital discharge notes, use of publicly funded home care services and date of death. For each patient, the relevant information was obtained for a period of 365 days after randomization.

Norway has a fully publicly funded health system, and these data should therefore cover any hospital admission, and any use of nursing home and home health care. The only exception is admissions to psychiatric hospitals, as this information is considered particularly sensitive and was not made available for the study. Mortality data in the registers are regularly synchronized with the official National Registry. The great majority of patients have records both in the community and hospital systems, allowing a cross-check of data in most cases.

### Changes to trial outcomes

Clinical observations during the trial period showed differences between medical and orthopaedic patient groups. After assessment of data we therefore decided to include subgroup analysis of these groups for the same outcome measures. This analysis was not pre-specified.

Designation of patients as either medical or orthopedic was based upon the main diagnosis on the hospital discharge note. The hospitals classify all diagnoses according to the tenth version of the World Health Organization’s International Classification of Diseases (ICD-10) [18]. Conditions categorized in ICD-10 chapters A00-B99, D50-D89, E00-E90, I00-I52, I70-I99, J00-J22, J30-J99, K00-K93, N00-N30 and N39 defined medical patients, while patients with conditions classified in ICD-10 chapters S00-T98 were defined as orthopedic.

### Sample size, generation of allocation sequence and type of randomization

A sample size of 400 patients was chosen in the first phase of the study, based upon previous studies [6, 19, 20] and data from similar patient groups in Scandinavian nursing homes (unpublished). The aim was to recognize an improvement of at least 10 % in functional outcome, with a strength of 80 % at a 5 % significance level, allowing a potential dropout rate of 30 % [15]. Although the second phase of the study had different endpoints, the sample size was still 400 patients as we examined the same population.

### Randomization

The random allocation sequence was computer-generated, using blocked randomization with a block size of 4 and allocation ratio of 1:1. Block randomization was chosen to prevent too much variability in number of patients randomized and ensure a reasonably steady flow of patients to each treatment group. A balance between intervention and control group for each center was secured by using a separate allocation sequence for each of the two hospitals. The sequence was available only for an independent study coordinator, based at a distance from both hospitals and the intermediate care facility. The study coordinator never met any of patients or staff, and was only reached by telephone. Sequence numbers was thus concealed from hospital staff and study investigators until interventions were assigned.

### Enrollment and assignment

At the two hospitals, doctors and nurses in medical and orthopedic emergency departments and wards were requested to consider every patient 70 year or older admitted from home. Decision to enroll patients was made by the attending physician after obtaining patient’s written consent. The doctor or nurse in charge of the patient made a telephone call to the independent study coordinator based outside the hospital, and patients were then assigned to intervention or control group according to the pre-specified allocation sequence. Blinding to group assignment was not possible. Blinding of outcome assessment in this second phase of the study would have been desirable. However, this was not feasible as the assessment involved reading the patients’ medical records from community health care and hospitals.

### Statistical methods

We collaborated with a professional and independent statistician, not otherwise involved with the study. Patients were analyzed as belonging to their original assigned “intention to treat” group. Statistical summaries and analyses were produced, using the software R [21] and SPSS version 18.0. Mann–Whitney U-test was used to compare groups for days living home, days in hospital and days in nursing home. Proportion test and chi-squared test was used to compare groups for mortality and independency of home care. Mortality was also analysed with Kaplan-Meier plot and log rank test. Similar tests were used for subgroup analyses of medical and orthopaedic patients.

### Ethics

Patients and caregivers were given oral and printed information and patients gave written consent before inclusion. Given careful selection of patients, the intervention was not regarded as potential harmful. Although the evidence base for intermediate care is limited, the service was already established. The Regional Committee for Medical Research Ethics approved the study.

## Results

### Participant flow

A total of 400 patients were randomized, 200 to intervention and 200 to control. During follow-up 24 patients changed their mind and withdrew consent. The resulting study population consisted of 376 patients, 190 in the intervention group and 186 in the control group. In the intervention group, 8 patients who were randomized to intermediate care needed to stay in hospital for medical reasons. All 190 patients were analyzed as belonging to the intervention group, in accordance with the “intention to treat” principle.

In the subgroup of medical patients, there were 78 patients in the intervention group and 72 in the control group. In the orthopedic subgroup there were 60 patients in the intervention group and 68 in the control group.

### Recruitment and follow-up

Recruitment of patients started 15^th^ August 2007 and was completed 2^nd^ June 2008. Enrolment ended when the number of included patients reached the pre-specified sample size of 400. For each patient data were obtained for a period of 365 days after randomization.

### Baseline data

Table 1 shows baseline characteristics for each group. In the study population of 376 patients, mean age was 84.1 years and 73.4 % were females. All patients were living at home prior to inclusion, 51.1 % with publicly funded home health care services of some degree. The most common primary diagnoses for medical patients were pneumonia, heart failure and chronic obstructive pulmonary disease. For orthopaedic patients the most common primary diagnoses were fracture of lumbar spine (compression fracture), minor pelvic fracture (pubic ramus fracture), rib fracture and contusions of lower back, pelvis or hip. Information whether patients were living alone was not registered systematically. Patients in the intervention group had a mean Barthel Index score of 70 (range 0–100) at arrival in the intermediate care unit. Of these, medical patients had a mean score of 80 (range 35–100) and orthopaedic patients 57 (range 0–100).

**Table 1:** **Baseline characteristics**

	Control group	Intervention group	p-value
Number of patients			
all patients	186	190	
medical patients	72 (38.7 %)	78 (41.1 %)	
orthopedic patients	68 (36.6 %)	60 (31.6 %)	
other	46 (24.7 %)	52 (27.3 %)	
Age, mean and range			
all patients	84.6 (71–98)	83.6 (70–96)	
medical patients	85.2 (72–98)	83.9 (70–96)	
orthopedic patients	83.9 (71–95)	84.0 (70–95)	
Proportion of females			
all patients	73.7 %	73.2 %	
medical patients	61.1 %	61.5 %	
orthopedic patients	82.4 %	85.0 %	
Proportion of patients with home health care			
all patients	53.2 %	48.9 %	0.47
medical patients	58.3 %	43.6 %	0.52
orthopedic patients	52.9 %	60.0 %	0.53
Barthel ADL Index score, mean and range			
all patients		70.0 (0–100)	
medical patients		80.0 (35–100)	
orthopedic patients		56.7 (0–100)	

### Numbers analysed

**Figure 1:**
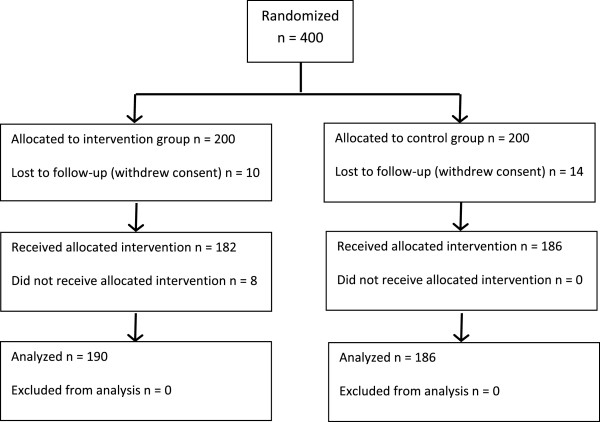
**Participant flow.**

Figure 1 shows the numbers of participants for each group who were randomly assigned, received intended treatment, and were analyzed for the primary outcome. Number of patients screened for eligibility was not consistently registered. Patients were only analyzed as part of their original assigned group, the “intention to treat” group. For the total study population there were 190 patients in the intervention group and 186 patients in the control group. For subgroup analyses of medical patients, there were 78 patients in the intervention group and 72 in the control group. For the orthopedic subgroup there were 60 patients in the intervention group and 68 in the control group.

### Outcomes and estimation

Length of stay in hospital before randomization was 1.6 days (range 0–4). Patients in the intervention group were transferred to Storetveit Nursing Home within one working day after randomization (mean 0.7 days, range 0–3), and stayed on average 17.3 days (range 1–34). At arrival in the intermediate care unit, intervention group patients had a mean Barthel Index score of 70 (range 0–100). Of these, medical patients had a mean score of 80 (range 35–100) and orthopaedic patients 57 (range 0–100). Two patients (1.1 %) died during the stay. At discharge, 75.8 % could return to their home, 17.6 % went to a nursing home and 5.5 % to other institutions or hospital.

Patients in the control group remained in hospital for a mean period of 7.0 days (range 0–36) after randomisation. One patient (0.5 %) died during the hospital stay. At discharge, 72.0 % could return to their home, 21.0 % went to a nursing home and 6.5 % to other institutions.

Main results are presented in Table 2. In the overall study population, there were no significant differences between groups for the primary outcome of days alive and living at home (253.7 vs 256.5, p = 0.80), or for number of days in hospital (10.4 vs 10.5, p = 0.748). There was however significant less use of nursing home in the intervention group (number of days in nursing home 40.6 vs. 55.0 days, p = 0.046), and more patients were independent from home health care services (31.6 % vs 19.9 %, p = 0.007). Relative risk for independence from home health care services was 1.59 (CI = 1.11 – 2.27) and mean number of days without home care was 27.5 days longer (97.7 vs. 70.2 days, p = 0.027). Mortality was increased in the intervention group (22.1 % vs. 17.2 %, RR 1.29), but the difference was not significant (p = 0.29, CI 0.85 – 1.94).

**Table 2:** **Results**

	Control group			Intervention group			p-value	Relative risk	Confidence interval	Relative effect size	Absolute effect size
	Mean	Range	SD	Mean	Range	SD					
Days living home											
all patients	256.5	0-364	125.1	253.7	0-359	120.4	0.80			÷ 1.1 %	÷ 2.8 days
medical patients	250.4	0-364	134.1	249.2	0-359	123.6	0.165			÷ 0.5 %	÷ 1.2 days
orthopedic patients	256.5	0-364	121.0	233.2	0-357	128.2	0.09			÷ 9.1 %	÷ 23.3 days
Days in nursing home											
all patients	55.0	0-360	91.7	40.6	0-344	71.4	0.046			÷ 26.1 %	÷ 14.4 days
medical patients	44.1	0-360	86.5	37.8	0-344	62.9	0.876			÷ 14.3 %	÷ 6.3 days
orthopedic patients	74.7	0-360	106.0	49.5	0-343	81.6	0.192			÷ 33.7 %	÷ 25.2 days
Days in hospital											
all patients	10.5	0-72	15.2	10.4	0-92	15.8	0.748			÷ 0.01 %	÷ 0.1 days
medical patients	12.9	0-72	17.2	10.6	0-70	14.9	0.530			÷18.1 %	÷ 2.3 days
orthopedic patients	8.2	0-61	12.7	12.0	0-92	19.0	0.536			+ 46.6 %	+ 3.8 days
One-year mortality											
all patients	17.2 %			22.1 %			0.29	1.29	0.85–1.94	+ 28.5 %	+ 4.9 %
medical patients	25.0 %			25.6 %			0.99	1.03	0.59–1.78	+ 2.4 %	+ 0.6 %
orthopedic patients	10.3 %			25.0 %			0.049	2.43	1.05–5.55	+ 142.7 %	+ 14.7 %
No home health care											
all patients	19.9 %			31.6 %			0.007	1.59	1.11–2.27	+ 58.8 %	+ 11.7 %
medical patients	18.1 %			35.9 %			0.011	1.99	1.12–3.53	+ 98.6 %	+ 17.8 %
orthopedic patients	19.1 %			30.0 %			0.219	1.57	0.84–2.93	+ 57.1 %	+ 10.9 %

### Ancillary analyses

**Figure 2:**
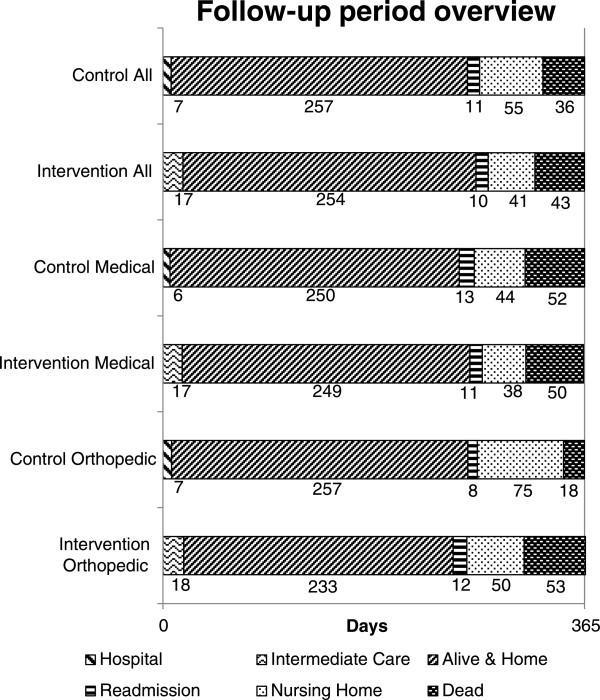
**Distribution of endpoints in different subgroups in the 365 days follow-up period (mean values).**

In subgroup analysis of orthopaedic patients, we found a significantly higher mortality in the intervention group (25.0 % vs. 10.3 %, p = 0.049). Relative risk for death was 2.43 (CI 1.05 – 5.55) and mean value for number of days alive was 35.0 days lower (311.9 vs. 346.9 days, p = 0.025).For medical patients number of days without home care was 52.0 days longer (97.2 vs. 53.5 days, p = 0.01), and relative risk for no home care services 1.99 (CI 1.12 – 3.53). To illustrate differences between groups, Figure 2 gives an overview of how many days (mean values) each patient group spent in hospital, in intermediate care, at home or in nursing home during follow-up.

## Discussion

### Main results

The findings suggest that patients treated in the intermediate care unit tended to be more independent, have a higher functional outcome and be better prepared for discharge. There was no effect on days living at home, but significant less need for home care services and nursing home.

### Limitations

There are several limitations of this study. To some degree these reflect that the intermediate care unit was planned and set up primarily with the objective to provide a new treatment option for elderly patients admitted to hospital. Organization of resources and patient flow was not necessarily optimized for the purpose of conducting a study. A key intention of the service was to achieve rapid assessment of patients, preferably in the emergency ward or the day after. Screening and selection of patients was by necessity carried out by the hospitals’ usual workforce of emergency nurses and doctors on duty. This put restrictions on selection and amount of information that could be gathered and registered. Opportunities to instruct and educate staff for this study in particular were also limited.

Partially for these reasons we do not have reliable information about number of patients initially screened for participation, and there is limited background data concerning proportion of patients living alone, and functional ability at baseline for control group patients. There is a theoretical possibility of important baseline differences between study groups; however the randomization should ideally prevent this. The study population was heterogeneous in degrees of functional ability, with Barthel ADL index score at baseline ranging from 0 to 100 in the intervention group. This can act as a limitation, masking or diluting beneficial or adverse effects in subgroups. Restrictions in available background data can make it difficult to compare the patient population in this study with other studies. On the other hand the diversity reflects real life situations in hospital emergency rooms and wards.

Selection of patients was based on fairly wide criteria regarding the medical condition causing hospital admission. Patients with delirium or severe cognitive impairment were supposed to be excluded, but adherence to this restriction was probably not absolute. Some doctors in the medical or orthopaedic wards would have limited experience in recognizing and assessing the conditions. Impetus to recruit patients would vary depending on the rate of admissions and demand for hospital beds. Estimation of patient suitability and ability to return home could therefore be influenced by the urge to discharge patients from overcrowded wards.

Before inclusion started, hospital staff was gathered and informed about the study and criteria for selecting patients. If in doubt doctors could call the geriatrician at the intermediate care unit and discuss patients before inclusion. However, selection of patients depended on doctors without formal geriatric training. The criterion “severe dementia” was not explicitly defined, and there was no formal instruction on how to identify or assess delirium or severe dementia. It’s therefore possible that selection criteria could be applied somewhat inconsistently, depending on different doctors’ experience and judgment.

There is also limited data regarding details of the hospital care for patients in the control group. Patients would generally receive appropriate medication like antibiotics and painkillers, and be discharged when clinically stable. In hospitals there would be a greater availability of diagnostic tests, especially radiologic tests, and technical monitoring equipment like telemetry, but a lesser degree of systematic multi-disciplinary assessment for each patient. The exact contents of “usual care” could in principle vary between the two hospitals and different departments in the same hospital.

Other limitations of the study include that the sample size was calculated for the original primary outcomes of functional outcome, quality of life and costs, and not for the re-defined primary outcomes of days living home. There is a possible risk of errors or omissions in the data sets obtained from community and hospital registers. The information we have accessed are the official records whereby all patients are obligatory registered when they are admitted to hospital or receive health care services from municipal authorities. Norway has a fully publicly funded health system which covers all citizens, and the medical records and registers should in principle be comprehensive and all-inclusive. Most patients had records both in the community and hospital, making a cross-check possible in the great majority of cases. Mortality data in the registers are regularly synchronized with the official National Registry. Admissions to psychiatric care were not recorded, but are believed to be rare. The randomization procedure should ensure potential errors to be evenly distributed between intervention and control groups.

### Interpretation

The results are to some extent diverging, as there seems to be both positive and negative effects of the intervention. There was overall a lack of effect on days living at home, but significant less need for home care services and nursing home. The findings suggest that patients treated in the intermediate care unit tended to be more independent, have a higher functional outcome and be better prepared for discharge.

When analyzing subgroups, the medical patients seemed to benefit most from this model of intermediate care. The intervention did not appear to be advantageous for the orthopaedic patients, with findings suggesting a higher mortality. This is a post-hoc analysis and therefore should not be given too much emphasis. Baseline data from the intervention group showed orthopedic patients to be frailer and have greater need for help in maintaining activities of daily living. Our findings could imply that frail patients admitted to hospital after acute trauma may not be suitable for this model of quick assessment and rapid transfer to a more basic treatment facility.

Our findings differ from previous studies of intermediate care in Norway which showed unequivocal benefit for patients [13, 14]. They are however comparable to the conclusions of a Cochrane review of nursing-led units where fewer patients were discharged to institutional care, but some studies showed a trend towards increased early mortality [9]. There is also a similarity to Young and coworkers’ study of post-acute care in community hospitals which showed that patients treated in intermediate care were more independent [7].

We expected the post-acute intermediate care to be advantageous for patients, based on previous studies and the fact that the service included principles of comprehensive geriatric assessment (CGA), known to be beneficial for hospitalized frail older people [17, 22, 23]. It can be argued that the Storetveit model with emphasis on early post-acute care is different from the Trondheim model, where randomization was 10 days after admission as opposed to 1.6 days at Storetveit. Also, the advantage of CGA is most evident for in-hospital geriatric evaluation and management units [17, 22, 23]. The intermediate care unit at Storetveit, though incorporating a geriatrician and a multidisciplinary team, had limited resources and could never be able to replicate the full scope of hospital-based CGA.

## Conclusions

This study indicates that some groups of older patients with acute illness can benefit from a post-acute care incorporating elements of comprehensive geriatric assessment. At the same time the results advices caution regarding the selection of frail patients to services with more limited resources than an ordinary hospital stay.

There is a need for more research to study different groups of patients, i.e. patients with or without cognitive impairment and medical vs. orthopaedic patients, identifying which patients are suited for intermediate care.

### Attribution

The work should be attributed to Kavli Research Centre for Geriatrics and Dementia, Haraldsplass Deaconess Hospital, Norway AND Department of Clinical Science, University of Bergen, Norway.
